# Phase Correction-Adaptive Line Enhancement for Noise Reduction of Low-Field NMR

**DOI:** 10.1007/s00723-013-0486-2

**Published:** 2013-11-02

**Authors:** Qingming Xie, Lizhi Xiao, Lijun Cheng, Junfeng Liu, Hongying Li, Feng Deng

**Affiliations:** 1 Key Laboratory of Shale Gas Exploration, Ministry of Land and Resources, Chongqing Institute of Geology and Mineral Resources, 177-9 Yangtze River 2 Road, Yuzhong, Chongqing, 400042 China; 2 Chongqing Shale Gas Research Centre of State Key Laboratory of Petroleum Resource and Prospecting, Chongqing, 400042 China; 3 Key Laboratory of Earth Prospecting and Information Technology, China University of Petroleum, Beijing, 102249 China

## Abstract

The amplitude of low-field nuclear magnetic resonance (NMR) is weak, and the echo is buried in the noise. The reduction of noise is critical to accurately extract echo amplitude. Phase correction-adaptive line enhancement (PC-ALE) is proposed to noise suppression based on the principle of ALE and NMR spin-echo characteristics. The echo amplitude is calculated after two-stage processes; phase shift from time-delay and filter tap would be compensated effectively in frequency domain. Simulation and experiments show that PC-ALE has prominent performance on noise suppression, envelope recovery, as well as the correction of the phase shift. The amplitude from the method of sample average nearby the middle of the echo is more accurate than the maximum peak when the PC-ALE is applied to noise suppression of spin-echo.

## Introduction

Low-field nuclear magnetic resonance (NMR) is applied for NMR well logging, core analyses, fluid magnetic resonance imaging, etc. [1, 2]. Compared to medium-field (0.500–1 T) and high-field (1.500–2 T) NMR [3], spin echo is easily contaminated by noise and the amplitude is weak. The reduction of noise is critical to accurately extract echo, the amplitude, and the phase information.

Methods of noise suppression in low-field NMR are diverse, such as, finite impulse response (FIR) filter [4], wavelet transform [5–7], principal component analyses (PCA) [8, 9], etc. FIR filter (i.e., low-pass, band-pass, and high-pass) is generally based on a priori knowledge of the spin echo; the de-noising performance is awful if a priori knowledge is insufficient.

The signal-to-noise ratio (SNR) is improved by accumulation [10]; however, the method requires a large amount of data acquisition and is a time-consuming procedure. Despite of the good performance on noise reduction, the wavelet transform and PCA are not suitable for the real-time process due to the computational complexity, and are adopted to post-processing of echo trains.

An adaptive noise canceller (ANC) based on the adaptive algorithm is widely used for noise reduction and weak signal extraction in recent years [11–15]. Adaptive line enhancement (ALE) is the degenerate form of ANC [11], and needs one input channel to detect the signal interfused by background noise, which depends on the principle of different autocorrelations between the signal and noise after a time delay. A large number of papers discuss the different adaptive algorithms for the NMR noise suppression [12–15]. In fact, the phase shift is a considerable factor for the NMR spectrum caused by the time delay of the ALE system, which results in the distortion of the position and amplitude of the NMR spectrum.

In this study, the two-stage adaptive de-noising with phase correction is proposed. Two-stage processes serve to reduce noise of the spin echo, and the phase correction in the frequency domain is achieved after the ALE process.

## Principle of PC-ALE

A spin echo can be described by

1 $$ x(t) = s(t) + v(t) = E\sin (2\pi ft + \phi_{0} ) + v(t), $$

where *E* represents the amplitude of the spin echo, *f* is the hydrogen nuclei precession frequency (called the Larmor frequency), $\phi_{0}$ is the initial phase, *v*(*t*) is the background noise. The noisy echo is filtered by a low-pass filter to eliminate high-frequency components, and is discreted by an analog–digital converter for digital processing.

**Fig. 1:**
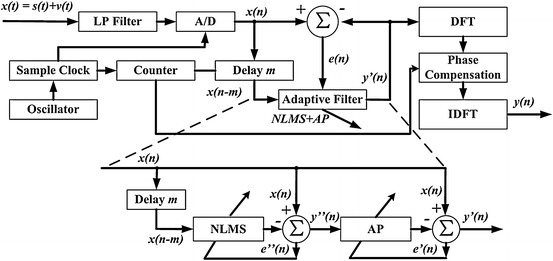
Principle of phase correction-adaptive line enhancement. *LP filter* low-pass filter, *A/D* analog to digital converter, *DFT* discrete Fourier transform, *IDFT* inverse discrete Fourier transform, *NLMS* normalized least mean square, *AP* affine projection

The PC-ALE system consists of two parts: noise reduction and phase correction, as shown in Fig. 1. The first part is crucial for accurately extracting the amplitude and the phase of the spin echo, which is a general ALE system including the discrete input data *x*(*n*) and its delay version. The delay *m* represents the prediction depth of the ALE. The input signal after delay *m* is processed by a transversal FIR filter and the output of the FIR filter *y*′(*n*) is given by

2 $$ y^{\prime}(n) = \sum\limits_{k = 0}^{L - 1} {W_{k} (n)x(n - k + 1)}, $$

where *W*_*k*_(*n*) represents the tap-weight of the adaptive filter at the time *n*, *L* is the filter order. The error signal *e*(*n*) is defined as the difference between the noisy echo and the ALE output. The error signal is employed to update the weights of the transversal filter with the adaptive algorithm. Development on adaptive algorithm is mainly based on Widrow and Hoff’s least-mean-square (LMS) algorithm [16]. The tap-weight of LMS is given by

3 $$ W(n + 1) = W(n) + \mu x(n)e(n), $$

where *e*(*n*) is the adaptation error at time *n*, *μ* is the fixed step size which is limited by $0 < \mu < 1/\lambda_{\hbox{max} }$, the $\lambda_{\hbox{max} }$ is the maximum eigenvalue of autocorrelation matrix for the input data *x*(*n*).

However, several significant elements for noise reduction of low-field NMR should be taken into account. First, the improvement of SNR is essential for the noisy spin echo. The accurate amplitude and phase information come from the high SNR data. Second, numerous computations from the matrix inversion of adaptive algorithm are not suited to fast measurement of low-field NMR tools. For example, the echo time of NMR well logging tools is generally few microseconds to measure the short relaxation of porous media that reflects the contents of clay bound water [17, 18]. Measurements are not precise if the computing time is larger than the echo time. Thirdly, the convergence rate and the stability are very important for the noise reduction of the spin echo.

With regard to the convergence rate, the variable step size has the better performance than the fixed step of LMS. The instantaneous mean square error can be minimized through the normalized least mean square (NLMS) algorithm without the evaluation of the matrix correlation [19, 20]. Moreover, the NLMS algorithm is stable with the low computational complexity. Another approach to improve the convergence rate is the affine projection (AP) algorithm [21–23], which is associated with the correlation of the spin echoes. The better SNR of signal, the faster convergence rate is. Consequently, both algorithms (NLMS and AP) are selected to the PC-ALE for the noise reduction of variety SNR data.

The NLMS algorithm is employed to the first-stage process for suppressing sine-shape noise superimposed on the spin echo. The correlation of the spin echo would be promoted as well. The step size of NLMS is given as follows

4 $$ \mu_{\text{NLMS}} (k) = 1/[2x^{\text{T}} (k)x(k)]. $$

The tap-weight adaptation of NLMS algorithm can be written as

5 $$ W_{\text{NLMS}} (k + 1) = W_{\text{NLMS}} (k) + [\mu_{\text{NLMS}} (k)e(k)x(k)]/[b + x^{\text{T}} (k)x(k)], $$

where *b* is a small constant that is used to avoid the excessive step size.

The AP algorithm is used to the second-stage process of ALE system, which transforms the data into the orthogonal structure, and makes the estimation of the tap-weight faster and more accurate. Step size and coefficients of AP are given as follows

6 $$ W_{\text{AP}} (k + 1) = W_{\text{AP}} (k) + \mu_{\text{AP}} x(k)[x^{\text{T}} (k)x(k)]^{ - 1} e(k)\,\quad 0 < \,\mu_{\text{AP}} < 2. $$

The second part is related to the phase correction. To verify the phase shift from the time-delay and filter tap, a linear correction algorithm can be performed. The algorithm is based on the fact that a delay in the time domain is equivalent to a phase shift in the frequency domain. Thanks to a priori knowledge of delay numbers of ALE system and the clock frequency, the phase shift can be dealt with in three steps as follows.

First of all, the spin-echo after the noise reduction is transformed from the time domain to the frequency domain with the discrete Fourier transform (DFT)

7 $$ Y^{\prime}(k) = {\text{DFT}}[y^{\prime}(n)] = \sum\limits_{n = 0}^{N - 1} {y^{\prime}(n)\exp \left( { - j\frac{2\pi }{N}nk} \right)} \quad \,k = 0, 1, \ldots ,N - 1;\;\;n = 0, 1, \ldots ,N - 1; $$

where *Y′*(*k*) denotes the spectrum of the NMR signal, *j* is the imaginary unit, *y′*(*n*) is the output of ALE system, *N* is the number of sampled data.

Moreover, the NMR spectrum in the frequency domain is multiplied by a correction factor

8 $$ Y(k) = Y^{\prime}(k) \times \exp \left( {j\frac{2\pi }{N}mk} \right), $$

where *m* is the delay number of the ALE system, it can be measured by a counter that operates on the clock signal the same as an analog-to-digital converter (ADC).

At last, *Y*(*k*) is inversely transformed to the time domain to give the corrected spin echo

9 $$ y(n) = {\text{IDFT}}[Y(k)] = \frac{1}{N}\sum\limits_{k = 0}^{N - 1} {Y(k)\exp \left( {j\frac{2\pi }{N}nk} \right)} . $$

The whole process of the PC-ALE outlines as follows: first, the narrow-band echo envelope and wideband noise are separated by the NLMS because of its excellent trace performance to sine wave, and correlation of spin-echo would be enhanced. Second, noise can be removed with the AP owing to the improvement of the spin echo correlation by the NLMS. Thirdly, the phase shift from the time delay and filter tap would be compensated in the frequency domain. At last, the spin echo in the frequency domain is transformed to the time domain with the inverse discrete Fourier transform.

## Numerical Simulation

Echo model is built for numerical simulation to noise reduction of low-field NMR. The amplitude of the echo envelope is assumed to 1 mV, Larmor frequency is 50 kHz, which corresponding to the static magnetic field strength is 1.174 × 10^−3^ T, sample frequency is 1 MHz, the SNR is −12.352 dB (the noise is with mean of zero). Here, SNR is given by

10 $$ {\text{SNR}} = 10\log_{10} (E_{\text{s}} /E_{\text{n}} ), $$

where *E*_s_ and *E*_n_ represent the energy of the signal and noise, respectively.

**Fig. 2:**
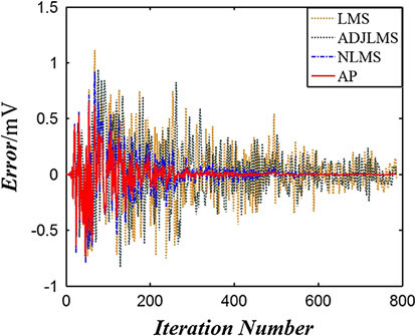
Comparison of error of a variety of algorithms. The fixed step size is 0.005, filter order is 32, and delay number is 3

As described in Sect. 2, both improvement of SNR and computation time of the algorithm should be considered due to the rigorous restriction of the short echo time. Several adaptive algorithms are compared for selecting the appropriate adaptive algorithms for noise reduction of the low SNR spin echo. The NLMS and AP algorithms are most appropriate adaptive algorithms for the PC-ALE according to the advantages of better SNR, faster convergence, and least computation, as shown in Fig. 2.

The convergence rate and error of four adaptive algorithms are from the noisy spin-echo model. Among these algorithms, the best convergence rate and error output are given by the AP algorithm, and the error is close to 0 after 220 iterations. NLMS exhibits sound performance to trace the sine wave, and is close to zero after 280 iterations. Convergence is achieved after 770 iterations for the LMS and ADJLMS [19], it is obviously not suitable for the noise reduction of the spin echo due to the slow convergence rate. The results are tabulated in Table 1, where BLMS [24] and DLMS [19] are the block LMS and delay LMS algorithms, respectively.

**Table 1:** Results of the noise reduction using a variety of algorithms

Algorithm	SNR (dB)	Time (s)	MA (mV)	SA-TE (mV)
Echo model			0.995	0.995
Noisy echo	−12.352		2.826	2.304
LMS^a^	−5.973	0.131	2.303	1.741
BLMS^a^	−5.999	0.036	2.240	1.712
ADJLMS^a^	−5.599	0.049	2.498	1.756
DLMS^a^	−5.998	0.032	2.299	1.512
NLMS^b^	−2.716	0.029	2.147	1.322
AP^a^	−2.217	0.072	2.216	1.385
NLMS^b^ + AP	1.054	0.109	1.928	1.223

^a^The fixed step size is 0.005, order is 32, delay number is 3

^b^The variable step size of the NLMS is Eq. (4)

SNR and computing time are listed in columns 2 and 3; NLMS is the most efficient algorithm as compared to the other adaptive algorithms. The computing time and the improvement of SNR are obviously better than those for the DLMS and ADJMS algorithms. In addition, the extreme increase in SNR can be achieved combining with the NLMS and AP, the increase of 13.406 dB in SNR can be implemented after the ALE system.

Two conventional methods for peak extractions of the spin-echo envelope are listed in columns 4 and 5. Column 4 is the maximum peak (MA) of the echo envelope after processing. Due to the noise preserved to peak, the difference of amplitude between noisy echo and model is relatively much larger. Column 5 represents the sample average near the middle of the echo (SA-ME). The maximum amplitude appears in the middle of the echo on the basis of the NMR principle regardless of the noise [1, 3]. However, the value of SA-ME is not equal to the MA value for the low SNR of the NMR spin echo because of the intense noise. The best result is the proposed two-stage processes (NLMS + AP) among the several algorithms. Compared to other algorithms, the MA and the SA-ME are close to the model after the PC-ALE processing.

**Fig. 3:**
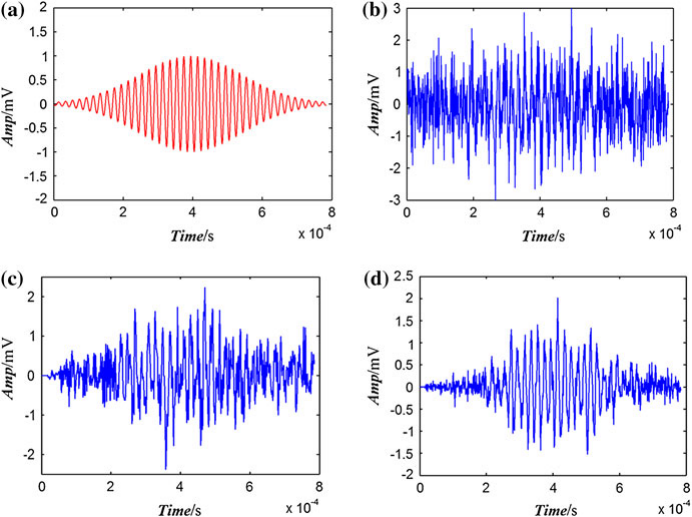
Model and noise reduction using PC-ALE. **a** Echo model, **b** noisy echo, **c** output of NLMS algorithm (first stage), **d** output of AP algorithm (second stage)

The results of double-stage processes are shown in Fig. 3. The clean spin echo is shown in Fig. 3a which splendidly reflects the NMR characteristic and 800 points are acquired. The amplitudes of MA and SA-ME are 0.995 mV. Figure 3b shows the noisy spin echo; the shape of the spin echo is covered by the noise and the envelope cannot be found because of the strongly random background noise. The MA and the SA-ME are 2.826 and 2.304 mV, respectively. Figure 3c is the first-stage process with NLMS algorithm, the SNR is −2.716 dB although the shape of the envelope is obscure. The MA and the SA-ME are 2.147 and 1.322 mV, respectively. Sample points from 600 to 800 still keep the background noise because the convergence of algorithm is implemented after 280 iterations, and the noise is processed as the sine signal. However, the correlation of the signal is enhanced after the noise suppression of the NLMS, which contributes to the next process. Figure 3d shows the second-stage processing with AP algorithm, the SNR is 1.054 dB and the shape of envelope is clear, the MA and the SA-ME are 1.928 and 1.223 mV, respectively.

However, there is a very important fact about the amplitude extraction with the PC-ALE algorithm. The difference between the MA and model are much larger than that for the SA-ME because the convergence rate is in conflict with the offset of the algorithm. When the convergence is implemented, a part of noise preserving to peak of the spin echo would be processed as signal, which leads to the increase in the offset. Furthermore, noise is a random distribution and is not related with each other. It can be decreased through averaging the several sample points near the middle of the echo. Consequently, the SA-ME plays the role of a smoothing filter; the amplitude for the SA-ME is more accurate than the maximum peak, and the SNR is improved as well. The amplitudes are close to that for the model after two-stage processes, and the noise is efficiently reduced by the ALE system.

**Fig. 4:**
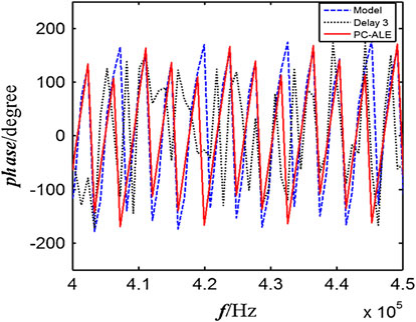
Phase correction using PC-ALE. The *blue dashed line* represents the phase of spin-echo model, *black dotted line* is the phase after delay three sample points, and *red line* means the phase correction using PC-ALE system (color figure online)

The phase shift in the frequency domain arises from the delay in the time domain. As a result, the NMR spectrum is distorted; both the amplitude and the position of the spin echo are strongly affected. The phase shift after ALE is corrected in the frequency domain with Eqs. (7)–(9). The results are shown in Fig. 4. The phase after correction is in good agreement with the original spin echo.

## Implementation of PC-ALE

**Fig. 5:**
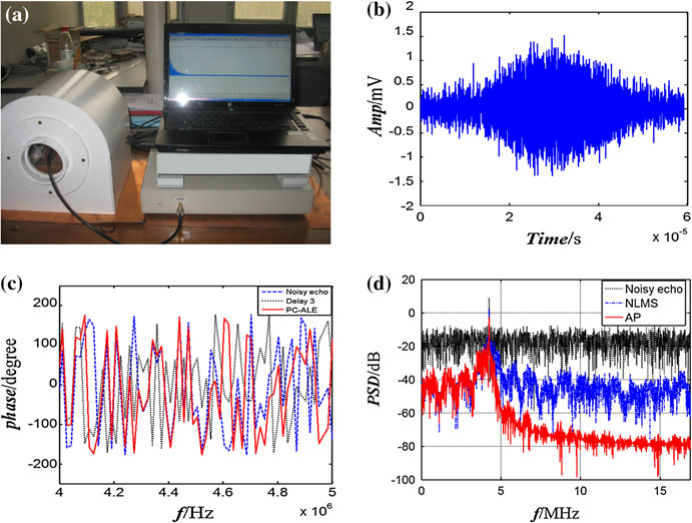
Power spectral density of noise reduction using PC-ALE. The sample is pure water. The adaptive filter order *N* = 32, the constant *b* = 1, step size of the AP algorithm $\mu_{\text{AP}}$ = 0.1; **(a)** Halbach magnet and the NMR digital spectrometer; **(b)** one of the spin-echoes collected by NMR spectrometer; **(c)** the phase of spin-echoes before and after the PC-ALE, the *blue dot line* is the phase of noisy echoes, the *red line* is the phase after noise reduction; **(d)** the power spectral density (PSD) of the spin-echo in different de-noising procedure, the *black dotted line* is the noisy echo, the *blue dashed line* is the PSD after NLMS algorithm, and the *red line* is the PSD after AP algorithm, the noise PSD decreases about −40 dB after the double-stage processes of PC-ALE

Spin echoes with different SNRs are acquired on a low-field NMR spectrometer made by our group to verify the effectiveness of PC-ALE. The experimental setup of a Halbach magnet and a low-field NMR spectrometer with a portable computer are depicted in Fig. 5a. The homogeneous static magnetic field strength is 0.100 T, and the radio frequency is 4.258 MHz. The sample frequency is 34.064 MHz, pulse width of 90° is 9.500 μs, pulse width of 180° is 19 μs,echo time is 400 μs, sample points are 2,400. The sample is pure water.

The PC-ALE algorithm is operated on the portable computer. Figure 5b shows one example of the noisy spin-echo acquired on the NMR spectrometer. The amplitude of the spin echoes is calculated with the methods of the MA and the SA-ME, as shown in Table 2. Spin echoes with the SNR from 5.899 to 14.228 are processed by PC-ALE. Despite of the amplitude reduction of the MA and the SA-ME, the SNR is improved after the noise suppression. The difference of phase after the PC-ALE is controlled within 3 degrees, because the phase is affected by many factors such as the inhomogeneous static magnetic fields, radio frequency shift, eddy currents from the coil in the surface of NMR sensor, instability of NMR spectrometer, et al. It is difficult to eliminate all of the factors completely, as shown in Fig. 5c. However, the purpose of this study is to focus on the phase shift from the time delay of adaptive filter processing; the other factors for phase shift would be considered in the next work. The power spectral density (PSD) of the spin-echo is calculated in the process of noise reduction using PC-ALE, as shown in Fig. 5d. The decrease in the noise PSD is about −40 dB after the double-stage processes. Obviously, noise is significantly reduced.

**Table 2:** Comparison of the noise reduction performance with PC-ALE

Number	Noisy spin echo				Output of PC-ALE			
	SNR (dB)	Peak (mV)	Half-TE (mV)	Phase (°)^a^	SNR (dB)	Peak (mV)	Half-TE (mV)	Phase (°)^a^
1	5.899	1.380	1.210	139.590	7.0604	1.351	1.198	140.249
2	5.663	1.373	1.064	135.809	6.879	1.133	1.063	133.652
3	8.412	2.009	1.836	173.190	22.544	1.819	1.692	175.897
4	9.043	1.510	1.177	−158.463	20.787	1.246	1.151	−161.199
5	9.559	2.032	1.465	−89.898	20.381	1.964	1.791	−87.550
6	9.092	2.094	1.781	28.678	22.087	1.869	1.680	27.365
7	9.033	1.818	1.724	−147.785	14.338	1.722	1.551	−146.204
8	11.275	1.938	1.678	13.774	21.382	1.837	1.688	12.286
9	12.998	2.035	1.857	128.137	22.571	1.991	1.794	129.202
10	14.228	1.855	1.521	126.703	15.368	1.811	1.642	123.922

^a^The phase are calculated in 4.258 MHz (the hydrogen nuclei precession frequency). The order is 32, delay number is 8

## Summary and Conclusions

Simulations and experimental investigations were used to evaluate the PC-ALE algorithm with various SNRs of spin echoes. The PC-ALE algorithm incorporates the advantages of the NLMS and the AP in the convergence rate and the correlation of the spin echo. The amplitude of the spin echo can be extracted accurately after two-stage processes of the PC-ALE, and the background noise keeping in the spin echo can be removed significantly. The phase shift from the time delay can be corrected in the frequency domain. The experiments show that PC-ALE has the prominent performance on the noise suppression, the envelope recovery, as well as the correction of the phase shift.

Moreover, the simulation shows that the amplitude calculation with SA-ME is more accurate than that with MA for the PC-ALE. The interference of the peak is decreased with the effect of the smoothing filter of the SA-ME. The phase shift is caused by multiple elements such as the inhomogeneous static field, the unstable radio frequency, the time delay, etc. Here, one factor is corrected by PC-ALE, others will be taken into account in a forthcoming work.
